# Quantification of Farnesylated Progerin in Hutchinson-Gilford Progeria Patient Cells by Mass Spectrometry

**DOI:** 10.3390/ijms231911733

**Published:** 2022-10-03

**Authors:** Emilio Camafeita, Inmaculada Jorge, José Rivera-Torres, Vicente Andrés, Jesús Vázquez

**Affiliations:** 1 Cardiovascular Proteomics Laboratory, Centro Nacional de Investigaciones Cardiovasculares Carlos III (CNIC), 28029 Madrid, Spain; 2 CIBER Enfermedades Cardiovasculares (CIBERCV), 28029 Madrid, Spain; 3 Molecular and Genetic Cardiovascular Pathophysiology Laboratory, Centro Nacional de Investigaciones Cardiovasculares Carlos III (CNIC), 28029 Madrid, Spain

**Keywords:** Hutchinson-Gilford progeria syndrome, progerin, farnesylation, mass spectrometry, targeted proteomics, precursor-reaction monitoring

## Abstract

Hutchinson-Gilford progeria syndrome (HGPS) is a rare fatal disorder characterized by premature aging and death at a median age of 14.5 years. The most common cause of HGPS (affecting circa 90% of patients) is a de novo heterozygous synonymous single-base substitution (c.1824C>T; p.G608G) in the LMNA gene that results in the accumulation of progerin, an aberrant form of lamin A that, unlike mature lamin A, remains permanently farnesylated. The ratio of progerin to mature lamin A correlates with disease severity in HGPS patients, and can be used to assess the effectiveness of therapies aimed at lessening aberrant splicing or progerin farnesylation. We recently showed that the endogenous content of lamin A and progerin can be measured by mass spectrometry (MS), providing an alternative to immunological methods, which lack the necessary specificity and quantitative accuracy. Here, we present the first non-immunological method that reliably quantifies the levels of wild-type lamin A and farnesylated progerin in cells from HGPS patients. This method, which is based on a targeted MS approach and the use of isotope-labeled internal standards, could be applied in ongoing clinical trials evaluating the efficacy of drugs that inhibit progerin farnesylation.

## 1. Introduction

**Figure 1:**
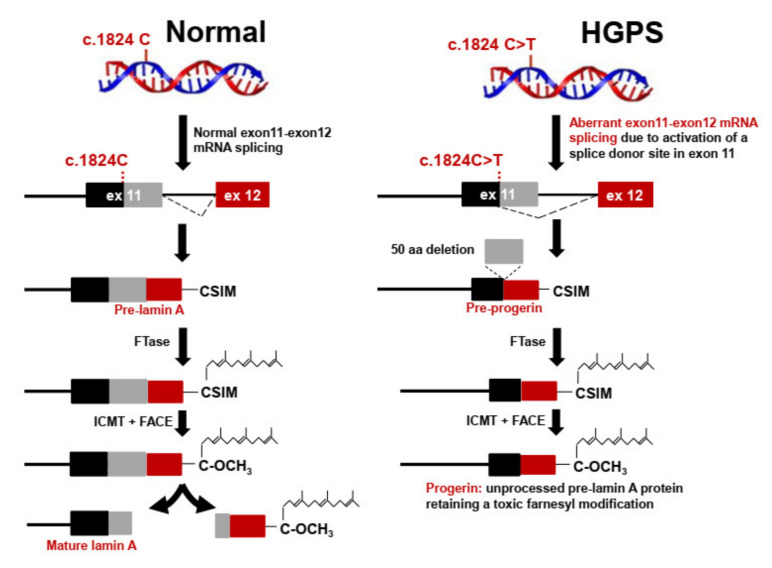
Defective post-translational processing of prelamin A in Hutchinson-Gilford progeria syndrome (HGPS). Newly synthesized prelamin A is farnesylated by farnesyltransferase (FTase) on the C-terminal CSIM motif. The CSIM motif is then cleaved by the Ras-converting CAAX endopeptidase 1 (RCE, also called FACE-2) or the zinc metalloprotease Ste24 (Zmpste24, also called FACE-1), and the resulting C-terminal Cys residue is methylated by the isoprenylcysteine carboxylmethyl transferase (ICMT). Finally, the 15 residues from the C-terminus, including the farnesylated and carboxymethylated C-terminal Cys, are cleaved by the endoprotease Zmpste24/FACE-1. In most HGPS patients, the mutation GGC to GGT in LMNA codon 608 (G608G) activates a cryptic splice site that causes the deletion of a 50-amino acid sequence containing the Zmpste24 cleavage site, thus preventing cleavage of the 15 C-terminal amino acid residues and resulting in the accumulation of the mutant form of farnesylated prelamin A called progerin.

Hutchinson-Gilford progeria syndrome (HGPS) is a systemic disease characterized by widespread atherosclerosis of the aorta and coronary and cerebral arteries, resulting in death at an average age of 14.5 years, predominantly from myocardial infarction or stroke [1,2]. Approximately 90% of HGPS patients have a de novo heterozygous synonymous mutation at codon 608 (c.1824C>T; p.G608G) in the LMNA gene that activates the use of an internal 5′ splicing site in exon 11 and results in the synthesis of progerin, an unprocessed, farnesylated form of prelamin A (Figure 1) [3,4]. Farnesylated progerin exerts a dominant-negative effect in cells and animal models of HGPS [5], and there is increasing evidence that disease severity and lifespan in progeria are largely determined by the total amount of farnesylated progerin and the ratio of progerin to mature lamin A [6,7]. While these findings paved the way to clinical trials with farnesyl transferase inhibitors (FTIs) [2,8,9], to date there have been no reports of reliable standardized methods for the quantification of the different lamin A proteoforms. Progerin is typically measured at the transcript level by RT-PCR and at the protein level by Western blot [6,10,11]. Severe drawbacks of immuno-based Western blotting include cross-reactivity, oligomer formation, long incubation times, poor quantitative accuracy, and an inability to achieve absolute quantification.

Since its discovery in the early 1990s, protein modification with isoprenoids has been the subject of many studies owing to its links to cancer, viral infection, and age-related disorders [12,13]. Protein prenylation (farnesylation and geranylgeranylation) has been investigated with a number of biochemical and molecular biology methods, most of them tailored to specific proteins. Early studies were aimed at identifying endogenously prenylated proteins and relied on the incorporation of radiolabeled isoprene derivatives into substrate proteins by FTase and geranylgeranyl transferases (GGTases) [14]. The disadvantages of this approach include low sensitivity, extremely long exposure times, and the need to handle radioactive material. To circumvent these limitations, alternative methods have since been developed that enable selective enrichment of prenylated proteins for subsequent immunological or MS-based detection; these methods include the use of fluorescent and biotin-tagged probes, as well as less sterically invasive biorthogonal reporter tags [15,16,17,18]. While these chemical-proteomics strategies have provided insight into protein prenylation in vitro and in living cells, only a limited number of human prenylated proteins have been conclusively identified with this approach.

The high sensitivity and wide dynamic range of liquid chromatography-tandem mass spectrometry (LC-MS/MS) has facilitated the unbiased direct analysis of many types of protein post-translational modification (PTM) [19], as well as the development of numerous bioanalytical assays for peptides or peptide analogues [20]. McClure et al. [21] proposed a complex procedure based on multiple reaction monitoring mass spectrometry (MRM-MS) to identify cysteine palmitoylation (another type of protein lipidation) in purified recombinant CFTR (cystic fibrosis transmembrane conductance regulator). Sorek et al. [22] used liquid phase extraction followed by gas chromatography-mass spectrometry (GC-MS) to identify farnesyl and geranylgeranyl moieties in the purified recombinant Arabidopsis Gγ subunits AGG1 and AGG2. Other studies have focused on members of the Ras protein superfamily, where endogenous prenylated peptides have escaped direct detection by MS [23,24]. Until very recently, direct MS-based analysis of endogenous prenylation has been confined to the simple mass measurement by matrix-assisted laser desorption/ionization (MALDI)-MS of farnesylated peptides from progerin [25] and guanine nucleotide-binding protein α-2 subunit [26] without unambiguous demonstration, based on MS/MS data, of the sequence and PTMs of the corresponding farnesylated peptides. Building on our recent finding that endogenous lamin A and progerin can be measured by MS [7], we demonstrate here that immune-based approaches could be replaced with a LC-MS/MS-based method for the quantification of lamin A and post-translationally farnesylated progerin in cells from HGPS patients.

## 2. Results and Discussion

### 2.1. Identification of Lamin A peptides by Shotgun LC-MS/MS

**Figure 2:**
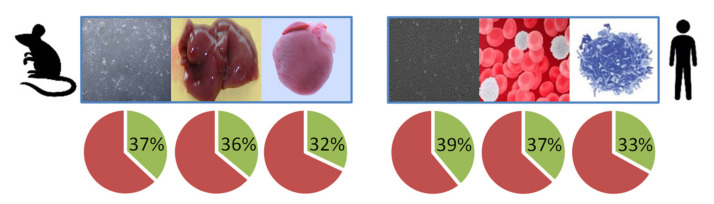
Sequence coverage of lamin A (mouse: UniProtKB accession number P48678, gene Lmna; human: UniProt KB accession number P02545, gene LMNA) in shotgun mass spectrometry (MS) experiments with mouse samples (skin fibroblasts and liver and heart tissue, left) and human samples (U2OS cells, white blood cells and lymphocytes, right). See Figure S1 for more detailed information.

**Figure 3:**
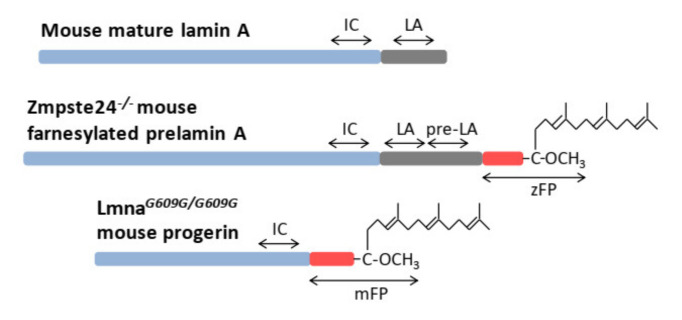
Schematic representation of the surrogate peptides selected for the measurement of mature lamin A, farnesylated prelamin A, and progerin in mouse samples: internal control peptide (*IC*), lamin A peptide (*LA*), prelamin A peptide (*pre-LA*), Zmpste24^−/−^ farnesylated prelamin A peptide (*zFP*), and Lmna*^609G/G609G^* mouse farnesylated peptide (*mFP*).

**Figure 4:**
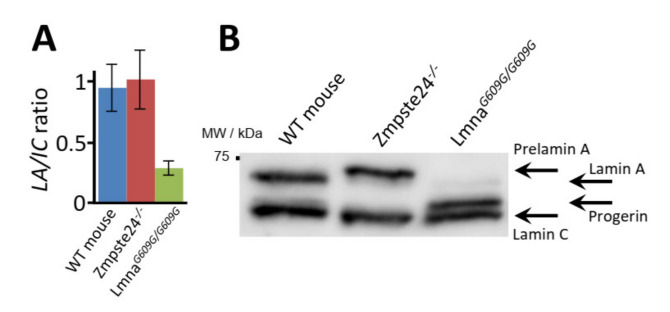
Detection of mature lamin A and progerin in skin fibroblasts from wild-type, Zmpste24*^−^*^/*−*^, and Lmna^609G/G609G^ mice. (**A**) *LA/IC* ratio obtained in shotgun mass spectrometry (MS) experiments. (**B**) Western blot analysis.

Shotgun LC-MS/MS analysis of a variety of mouse and human cell and tissue samples yielded >30% sequence coverage of lamin A (Lmna gene, UniProtKB entry P48678), including residues spanning the 50-amino acid region deleted in HGPS (Figure 2 and Figure S1). Considering these results together with the amino acid sequences of mouse prelamin A, mature lamin A, and progerin, we selected surrogate peptides for the targeted quantification of these proteoforms in cultured fibroblasts from wild-type mice and from progeroid homozygous Zmpste24^−/−^ and Lmna*^G609G/G609G^* mice, which accumulate prelamin A and progerin, respectively (Figure 3). The peptide sequences were as follows: TVLCGTCGQPADK, the internal control peptide (*IC*), common to mouse prelamin A, lamin A, farnesylated prelamin A, and progerin; SYLLGNSSPR, the prelamin A signature peptide (*pre-LA*), unique to farnesylated prelamin A, which accumulates in Zmpste24^−/−^ mice; SVGGSGGGSFGDNLVTR, the prelamin A/lamin A signature peptide (*LA*), unique to mouse prelamin A and lamin A; SQSSQNC, the O-methylated, farnesylated Cys signature peptide unique to Zmpste24^−/−^ mouse farnesylated prelamin A (*zFP,* which accumulates in Zmpste24^−/−^ mice); and AAGGAGAQSSQNC, the O-methylated, farnesylated Cys signature peptide unique to Lmna*^G609G/G609G^* mouse farnesylated progerin (*mFP,* which accumulates in Lmna*^G609G/G609G^* mice). In a first approach, we detected the *LA* and *IC* peptides with a shotgun (i.e., non-targeted) analysis of the skin fibroblast nuclear fractions from the three mouse strains. On the basis of the corresponding extracted ion chromatograms (XICs), we estimated the corresponding *LA*/*IC* ratio (Figure 4A). As expected, the *LA*/*IC* ratio was circa 1 in fibroblast nuclei from wild-type and Zmpste24^−/−^ mice, but was about three-fold lower in knock-in Lmna*^G609G/G609G^* mice. The relative abundance of prelamin A, mature lamin A, and progerin measured by Western blot is consistent with these *LA*/*IC* ratio values (Figure 4B). However, this shotgun approach did not detect the farnesylated peptides *zFP* and *mFP*.

### 2.2. Relative Quantification of Lamin A and Progerin in Mice Mouse Fibroblasts by Parallel Reaction Monitoring

**Figure 5:**
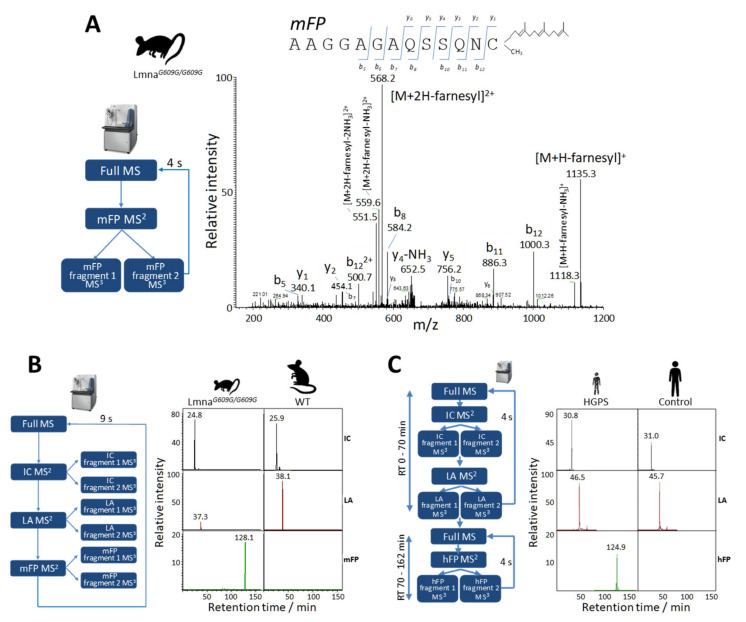
Relative quantification of progerin by parallel reaction monitoring (PRM) analysis in mouse and human samples. (**A**) MS^2^ fragmentation spectrum of the endogenous *mFP* farnesylated peptide obtained by PRM analysis of skin fibroblast nuclei from progeroid Lmna^609G/G609G^ mice. The chart shows ion ascription to the main fragment-ion series (C-terminal y-series and N-terminal b-series). (**B**) Representative MS^3^ extracted ion chromatograms (XICs) of *IC*, *LA*, and *mFP* peptides obtained from the PRM assay with Lmna^G609G/G609G^ and wild-type mouse skin fibroblasts for the relative quantification of lamin A and progerin (see Table 1 for details). Relative intensities were normalized to that of *IC*, which was assigned an arbitrary intensity of 100 in the Lmna^609G/G609G^ sample. (**C**) Representative MS^3^ XICs of *IC*, *LA* and *hFP* peptides obtained by time-scheduled PRM assay with human Hutchinson-Gilford progeria syndrome (HGPS) and control skin fibroblasts for the relative quantification of lamin A and progerin (see Table 2 for details). Relative intensities were normalized to that of endogenous peptide *IC*, which was assigned an arbitrary intensity of 100 in the HGPS sample.

Given the inability of the shotgun approach to identify *zFP* and *mFP* (the surrogate peptides for prelamin A and progerin, respectively), we sought to detect them with parallel reaction monitoring (PRM) assays. This approach eventually succeeded in detecting both peptides, first in the nuclear fraction of skin fibroblasts (Figure 5A and Figure S2, respectively) and later in the corresponding total protein extract (data not shown). We then conducted a PRM assay with the surrogate peptides relevant to the relative quantification of lamin A and progerin in Lmna^G609G/G609G^ mice, namely *IC*, *LA*, and *mFP* (Table 1). The total protein extracts from Lmna*^G609G/G609G^* mouse skin fibroblasts contained a very low amount of lamin A and quantifiable amounts of progerin, contrasting with wild-type fibroblasts, which, as expected, contained no detectable trace of progerin (Figure 5B).

### 2.3. Relative Quantification of Lamin A and Progerin in Human Fibroblasts by PRM

An optimized, time-scheduled PRM assay was then designed for the relative quantification of lamin A and progerin in human samples (Table 2). The analysis of total protein extracts from HGPS patient skin fibroblasts revealed a decreased level of lamin A and quantifiable amounts of progerin, contrasting with control individuals, in whom no trace of progerin was detected (Figure 5C). To our knowledge, this is the first report of the direct identification of the farnesylated peptide from human progerin.

### 2.4. Absolute Quantification of Lamin A and Progerin in Human Samples by PRM

**Figure 6:**
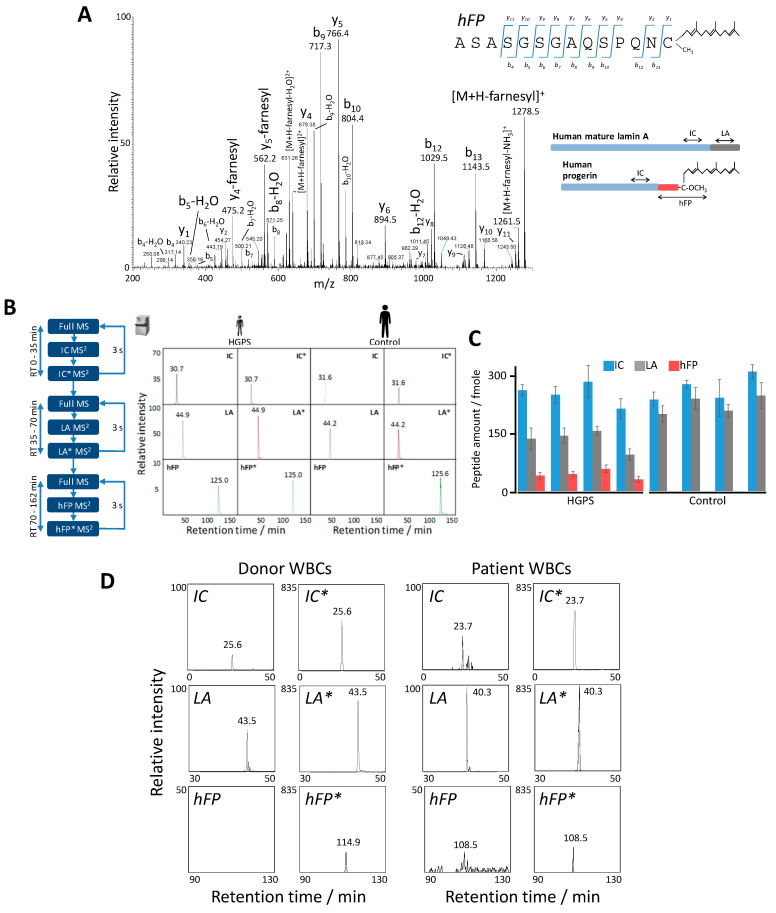
Absolute quantification of human progerin by parallel reaction monitoring (PRM) analysis. (**A**) Representative MS^2^ fragmentation spectrum of the endogenous, farnesylated *hFP* peptide obtained in the PRM assay (see scheme) from skin fibroblasts of Hutchinson-Gilford progeria syndrome (HGPS) patients. The inset shows the ion ascription to the main fragment-ion series (C-terminal y-series and N-terminal b-series). (**B**) Representative MS^2^ XICs of endogenous *IC*, *LA*, and *hFP* (see scheme in (**A**)) and the isotope-labeled, spiked-in peptides IC*, LA*, and hFP* obtained in the time-scheduled PRM assay with human HGPS and control skin fibroblasts for the absolute quantification of lamin A and progerin (see Table 3 for details). (**C**) Absolute quantification of surrogate peptides *IC*, *LA*, and *hFP* in four human HGPS and four control fibroblast samples. Error bars indicate standard deviation for triplicate analysis. (**D**) MS^2^ XICs of surrogate peptides *IC*, *LA*, *hFP*, and the isotope-labeled, spiked-in peptides IC*, LA*, and hFP* measured in the PRM assay with WBCs from a healthy donor (Left) and an HGPS patient (Right). Relative intensities were normalized to that of endogenous peptide *LA*, which was assigned an arbitrary intensity of 100 in the patient sample, except for the *hFP* peptide, which was scaled to 50. Retention time deviations between donor and patient are due to the use of different chromatographic columns (see Supplementary Methods).

To achieve absolute quantification of human lamin A and progerin, the tryptic digests of total protein extracts from four human HGPS and four control fibroblast samples were spiked with controlled amounts of the synthetic, isotopically labeled versions of the corresponding surrogate peptides and assayed in triplicate by time-scheduled PRM analysis (Table 3). By matching the XICs from endogenous *IC*, *LA*, and *hFP* surrogate peptides to those from exogenous IC*, LA*, and hFP* synthetic peptides (Figure 6A,B), we obtained absolute quantification of lamin A and progerin in samples from human HGPS patients and control individuals, with coefficients of variation below 20% in most cases (Figure 6C). As expected, progerin was undetectable in control human fibroblasts. In contrast, human HGPS fibroblasts contained on average 43 ± 8 fmole of progerin per 100 μg of protein extract, and this was accompanied by 133 ± 19 fmole of lamin A per 100 μg of protein extract, versus 224 ± 26 fmole in control samples. It must be taken into account that, since the usage of peptide standards does not allow to control peptide losses upon peptide extraction from the gel matrix, this method cannot provide a true absolute quantification. Notwithstanding this limitation, the PRM assay described provides an invaluable means of monitoring therapeutic efficacy and correlating progerin content to disease severity and progression.

### 2.5. PRM Analysis of of Lamin A and Progerin in Blood from HGPS Patients

While the procedure described above allows the absolute quantification of lamin A and progerin, the HGPS patient fibroblasts were obtained through an invasive procedure—a superficial punch skin biopsy collected under local anesthesia. It is, therefore, vitally important to develop a non-invasive, MS-based assay able to quantify lamin A and progerin in HGPS patients with high sensitivity and specificity. Since white blood cells (WBCs) can be easily isolated from routine blood-test samples, we sought to detect lamin A and progerin in WBCs from HGPS patients. Tryptic digests were prepared from WBCs isolated from control and HGPS blood samples, and the resulting peptide samples were spiked with known amounts of the synthetic, isotopically labeled surrogate peptides used to quantify lamin A and progerin (Table 3). These peptide mixtures were subjected to the same time-scheduled PRM analysis used in the previous subsection. The endogenous peptides *IC* and *LA* were successfully detected in WBCs from both HGPS patients and healthy donors (Figure 6D); interestingly, endogenous *hFP* was unambiguously identified in WBCs from HGPS patients (although below the quantification limit), whereas, as expected, no traces of this peptide were detected in control samples. This finding paves the way to the development of a non-invasive proteomics assay for measuring progerin in blood samples from HGPS patients. Work is currently ongoing to make necessary improvements to develop this assay.

## 3. Materials and Methods

Detailed Materials and Methods can be found in the Supplementary Methods file.

### 3.1. Biological Materials

#### 3.1.1. Mouse Cells and Tissues

Adult mouse skin fibroblasts were isolated from 15-week-old wild-type mice and from progeroid homozygous Zmpste24^−/−^ and Lmna*^G609G/G609G^* mice, which phenocopy the main clinical manifestations of human HGPS, including cardiovascular aberrations and premature death. Mouse liver and heart tissue samples were taken from 15-week-old wild-type mice [27,28].

#### 3.1.2. Human Cells

Primary human skin fibroblasts from four healthy individuals and four HGPS patients were obtained from the Progeria Research Foundation Cell and Tissue Bank. Human osteosarcoma U-2 OS cells (Sigma-Aldrich, Darmstadt, Germany) and human Jurkat-derived J77 (TCR Vαl.2 Vβ8) T-cells were used. Human WBCs isolated from three healthy individuals and three HGPS patients were provided by the Progeria Research Foundation Cell and Tissue Bank.

### 3.2. Synthetic Peptides

Three synthetic peptides (JPT Peptide Technologies, Berlin, Germany) were used in the PRM assay aimed at the absolute quantification of lamin A and progerin in human samples (second point from Section 3.5.2):

- Heavy internal control peptide, IC* (TVLCGTCGQPADK);
- Heavy lamin A peptide, LA* (SVGGSGGGSFGDNLVTR);
- Heavy human progerin farnesylated peptide, hFP* (ASASGSGAQSPQNC, with O-methylated, farnesylated Cys).

### 3.3. Preparation of Protein Extracts

#### 3.3.1. Protein Extracts for LC-MS/MS Analysis

Nuclear fractions were prepared from whole-cell pellets using a non-ionic detergent-based procedure [29]. Frozen mouse tissues were ground with a mortar and further homogenized with a Polytron tissue grinder. Tissue, whole-cell, and nuclear protein extracts were prepared by boiling in high-SDS lysis buffer for 5 min. Lysates were then centrifuged, and the protein concentration in supernatants was measured with the RC/DC protein assay kit (BioRad, Hercules, CA, USA).

#### 3.3.2. Protein Extracts for Western Blot Analysis

Cell extracts were prepared in low-SDS lysis buffer, and protein concentration was determined by Bradford assay, using BSA as a standard (Protein Assay Kit, Bio-Rad).

### 3.4. Protein Digestion

The protein extracts obtained in Section 3.3.1 were trypsin-digested separately using a one-step in-gel digestion protocol described previously [30]. The resulting peptide mixtures were desalted in RP C-18 extraction cartridges (Oasis, Waters, Milford, MA, USA) and dried down.

### 3.5. LC-MS/MS Analysis

#### 3.5.1. Shotgun LC-MS/MS Analysis

High-resolution shotgun analysis of peptides was performed with an Ultimate 3000 nano-HPLC apparatus (Dionex, Sunnyvale, CA, USA) coupled to an orbital ion trap mass spectrometer (LTQ-Orbitrap XL, Thermo Scientific, San Jose, CA, USA).

#### 3.5.2. Targeted PRM LC-MS/MS Assays

##### Targeted PRM LC-MS/MS Assay for the Relative Quantification of Lamin A and Progerin in Mouse Samples

Targeted LC-MS/MS analysis of mouse samples was performed by high-resolution PRM with an Ultimate 3000 nano-HPLC apparatus (Dionex) coupled to an orbital ion trap mass spectrometer (LTQ-Orbitrap XL, Thermo Scientific).

##### Targeted PRM LC-MS/MS Assays for Relative or Absolute Quantification of Lamin A and Progerin in Human Samples

Human samples spiked with the heavy synthetic peptides IC*, LA*, and hFP* (Section 3.2) were analyzed by high-resolution PRM using an Easy nLC 1000 nano-HPLC apparatus (Thermo Scientific) coupled to a hybrid ion trap-orbitrap mass spectrometer (Orbitrap Elite, Thermo Scientific).

### 3.6. Protein Identification and LC-MS/MS Data Analysis

Proteins were identified from shotgun LC-MS/MS scans using the SEQUEST HT algorithm integrated in Proteome Discoverer 1.4 (Thermo Scientific). For peptide identification, the probability ratio method was used [31], and the false discovery rate (FDR) of peptide identifications was calculated by the refined method [32]. Xcalibur 2.2 (Thermo Fisher Scientific) was used to obtain the extracted ion chromatograms (XICs) of selected ion fragments from PRM scans.

### 3.7. Western Blot Analysis

The protein extracts obtained in Section 3.3.2 were mixed with Laemmli sample buffer and resolved on 10% SDS–polyacrylamide gels. Proteins were then transferred to PVDF membranes. The membranes were incubated for 1 h at room temperature in blocking buffer, followed by overnight incubation at 4 °C with primary antibodies diluted in blocking buffer. After washing, the membranes were incubated with HRP-conjugated secondary antibodies (Santa Cruz Biotechnology, Dalla, TX, USA) for 1 h at room temperature. Specific proteins were then visualized by enhanced chemiluminescence (GE Healthcare, Chicago, IL, USA).

## 4. Conclusions

We report here the first method that reliably quantifies the absolute levels of lamin A and farnesylated progerin in cells from HGPS patients with high sensitivity and specificity. The method is based on the PRM LC-MS/MS analysis of surrogate peptides for lamin A and farnesylated progerin, and heavy derivatives of these, in fibroblasts from HGPS patients. A less invasive assay to quantify lamin A and progerin in HGPS-patient blood is still under development; nevertheless, the evidence presented here establishes the feasibility of detecting these proteins in blood. We believe that this method will prove to be an invaluable tool for monitoring patients treated with drugs aimed at inhibiting progerin farnesylation.

## Figures and Tables

**Table 1:** Precursor and fragment ions traced in the targeted PRM LC-MS/MS analysis of Lmna*^G609G/G609G^* mouse samples.

Symbol	Description	Amino Acid Sequence	MS^2^ m/z (Charge)	MS^3^ m/z (Ion)	MS^3^ XIC Trace
*IC*	Internal control peptide	TVLC^#^GTC^#^GQPADK	703.8238 (+2)	430.2296 (y_4_)	430.2296 > 333.1769 (y_3_) + 603.7657 > 933.4095 (y_9_)
				603.7657 (y_11_^2+^)	
*LA*	Mature lamin A peptide	SVGGSGGGSFGDNLVTR	783.8791 (+2)	690.8288 (y_15_^2+^)	690.8288 > 774.4104 (y_7_) + 774.4104 > 602.3620 (y_5_)
				774.4104 (y_7_)	
*mFP*	Lmna*^G609G/G609G^* mouse farnesylated peptide	AAGGAGAQSSQNC^Δ,O^	670.3374 (+2)	568.2435 (NL^2+^)	568.2435 > 886.4013 (b_11_) + 1135.4797 > 993.4054 (y_11_)
				1135.4797 (NL)	

C#, carbamidomethylated Cys; C^Δ,O^, farnesylated, O-methylated Cys; LC-MS/MS, liquid chromatography-tandem mass spectrometry; m/z, mass-to-charge ratio; NL, farnesyl group neutral loss; PRM, precursor-reaction monitoring; XIC, extracted ion chromatogram.

**Table 2:** Precursor and fragment ions included in the PRM assay aimed at the relative quantitation of lamin A and progerin in human samples.

RT Range	Symbol	Description	Amino Acid Sequence	MS^2^ m/z (Charge)	MS^3^ m/z (Ion)	MS^3^ XIC Trace
0–70 min	*IC*	Internal control peptide	TVLC^#^GTC^#^GQPADK	703.8238 (+2)	430.2296 (y_4_)	430.2296 > 333.1769 (y_3_) + 603.7657 > 933.4095 (y_9_)
					603.7657 (y_11_^2+^)	
	*LA*	Mature lamin A peptide	SVGGSGGGSFGDNLVTR	783.8791 (+2)	690.8288 (y_15_^2+^)	690.8288 > 774.4104 (y_7_) + 774.4104 > 602.3620 (y_5_)
					774.4104 (y_7_)	
70–162 min	*hFP*	Human farnesylated peptide	ASASGSGAQSPQNC^Δ,O^	741.8665 (+2)	639.7726 (NL^2+^) 1278.5379 (NL)	639.7726 > 717.3162 (b_9_) + 1278.5379 > 1260.5273 (-H_2_O)

C^#^, carbamidomethylated Cys; C^Δ,O^, farnesylated, O-methylated Cys; m/z, mass-to-charge ratio; NL, farnesyl group neutral loss; PRM, precursor-reaction monitoring. RT, retention time; XIC, extracted ion chromatogram.

**Table 3:** Precursor ions traced in the PRM assay aimed at the absolute quantitation of lamin A and progerin in human samples.

RT Range	Symbol	Description	Amino Acid Sequence	MS^2^ m/z (Charge)	MS^2^ XIC Trace
0–35 min	*IC*	Internal control peptide	TVLC^#^GTC^#^GQPADK	703.8238 (+2)	703.8238 > 933.4095 (y_9_) + 703.8238 > 603.7657 (y_11_^2+^)
	IC*	Isotopically labeled *IC*	TVLC^#^GTC^#^GQPADK^*^	707.8309 (+2)	707.8309 > 941.4237 (y_9_) + 707.8309 > 607.7728 (y_11_^2+^)
35–70 min	*LA*	Mature lamin A peptide	SVGGSGGGSFGDNLVTR	783.8791 (+2)	783.8791 > 690.8288 (y_15_^2+^) + 783.8791 > 921.4789 (y_8_)
	LA*	Isotopically labeled *LA*	SVGGSGGGSFGDNLVTR^*^	788.8831 (+2)	788.8831 > 695.8329 (y_15_^2+^) + 788.8831 > 931.4871 (y_8_)
70–162 min	*hFP*	Human farnesylated peptide	ASASGSGAQSPQNC^Δ,O^	741.8665 (+2)	741.8665 > 1278.5379 (NL) + 741.8665 > 1029.4596 (b_12_)
	hFP*	Isotopically labeled *hFP*	A^*^SASGSGAQSPQNC^Δ,O^	743.8700 (+2)	743.8701 > 1286.5521 (NL) + 743.8701 > 1033.4667 (b_12_)

A^*^, 3 x ^13^C + 1 x ^15^N N-terminal Ala; C^#^, carbamidomethylated Cys; C^Δ,O^, farnesylated and O-methylated Cys; K^*^, 6 x ^13^C + 2 x ^15^N Lys; m/z, mass-to-charge ratio; NL, farnesyl group neutral loss; PRM, precursor-reaction monitoring; R^*^, 6 x ^13^C + 4 x ^15^N Arg; RT, retention time; XIC, extracted ion chromatogram.

## Data Availability

The LC-MS/MS raw files used for the quantification of lamin A and progerin are available in the jPOST repository (https://repository.jpostdb.org/) under accession number JPST001825 (accessed on 26 August 2022).

## Supplementary Materials

The supporting information can be downloaded at https://www.mdpi.com/article/10.3390/ijms231911733/s1.
