# Supplementary material for: Quantification of Farnesylated Progerin in Hutchinson-Gilford Progeria Patient Cells by Mass Spectrometry
Source: Int J Mol Sci. 2022 Oct 3;23(19):11733. doi: 10.3390/ijms231911733 (PMC9569443; doi:10.3390/ijms231911733)
Supplement: Supplementary file 1 [file ijms-23-11733-s001.zip › ijms-1916264-supplementary/Figure S1.pptx]

## Slide 1
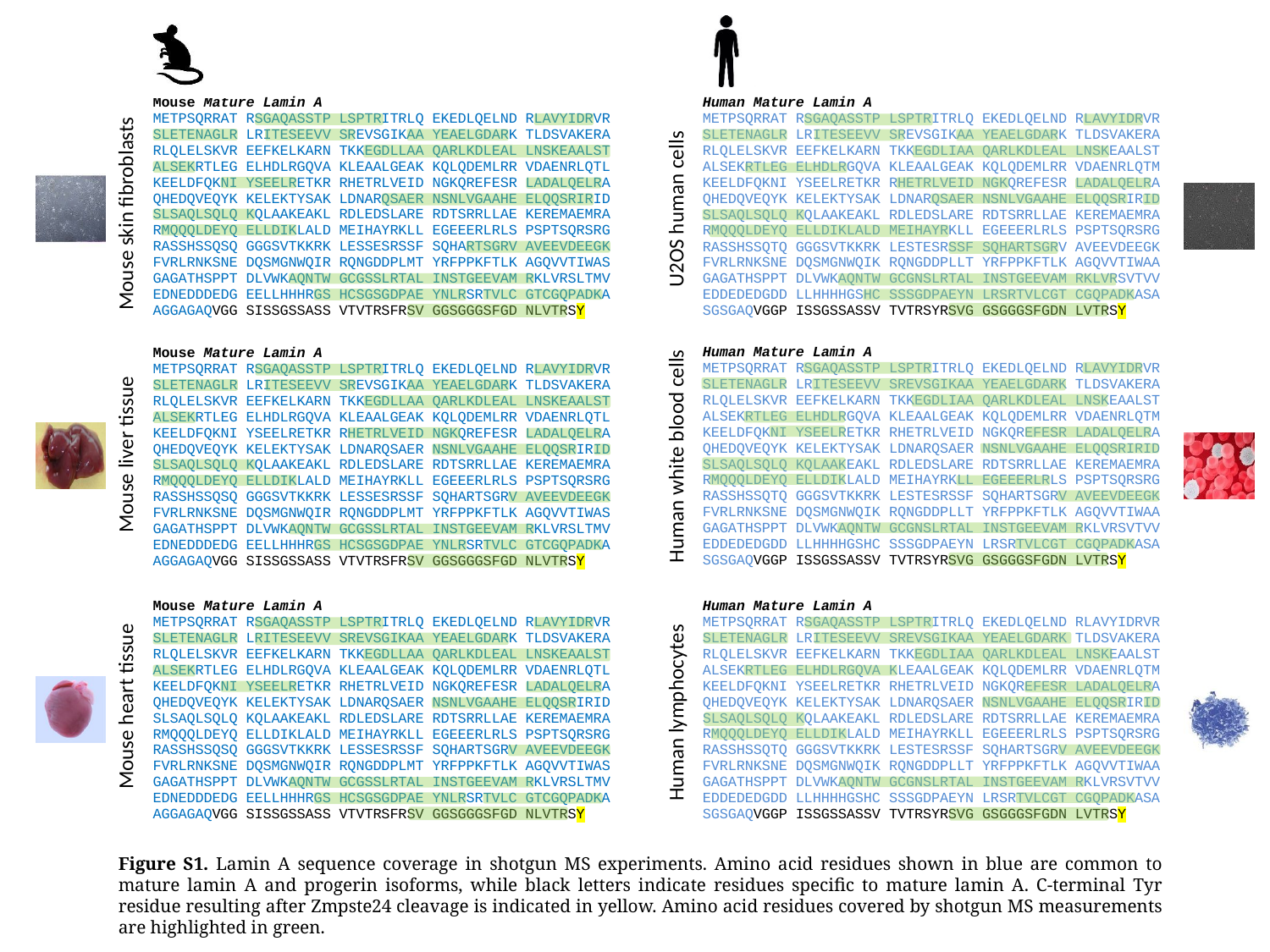

U2OS human cells
Mouse skin fibroblasts
Mouse liver tissue
Human white blood cells
Mouse heart tissue
Human lymphocytes
Figure S1. Lamin A sequence coverage in shotgun MS experiments. Amino acid residues shown in blue are common to mature lamin A and progerin isoforms, while black letters indicate residues specific to mature lamin A. C-terminal Tyr residue resulting after Zmpste24 cleavage is indicated in yellow. Amino acid residues covered by shotgun MS measurements are highlighted in green.
