# Supplementary material for: Quantification of Farnesylated Progerin in Hutchinson-Gilford Progeria Patient Cells by Mass Spectrometry
Source: Int J Mol Sci. 2022 Oct 3;23(19):11733. doi: 10.3390/ijms231911733 (PMC9569443; doi:10.3390/ijms231911733)
Supplement: Supplementary file 1 [file ijms-23-11733-s001.zip › ijms-1916264-supplementary/Figure S2.pptx]

## Slide 1
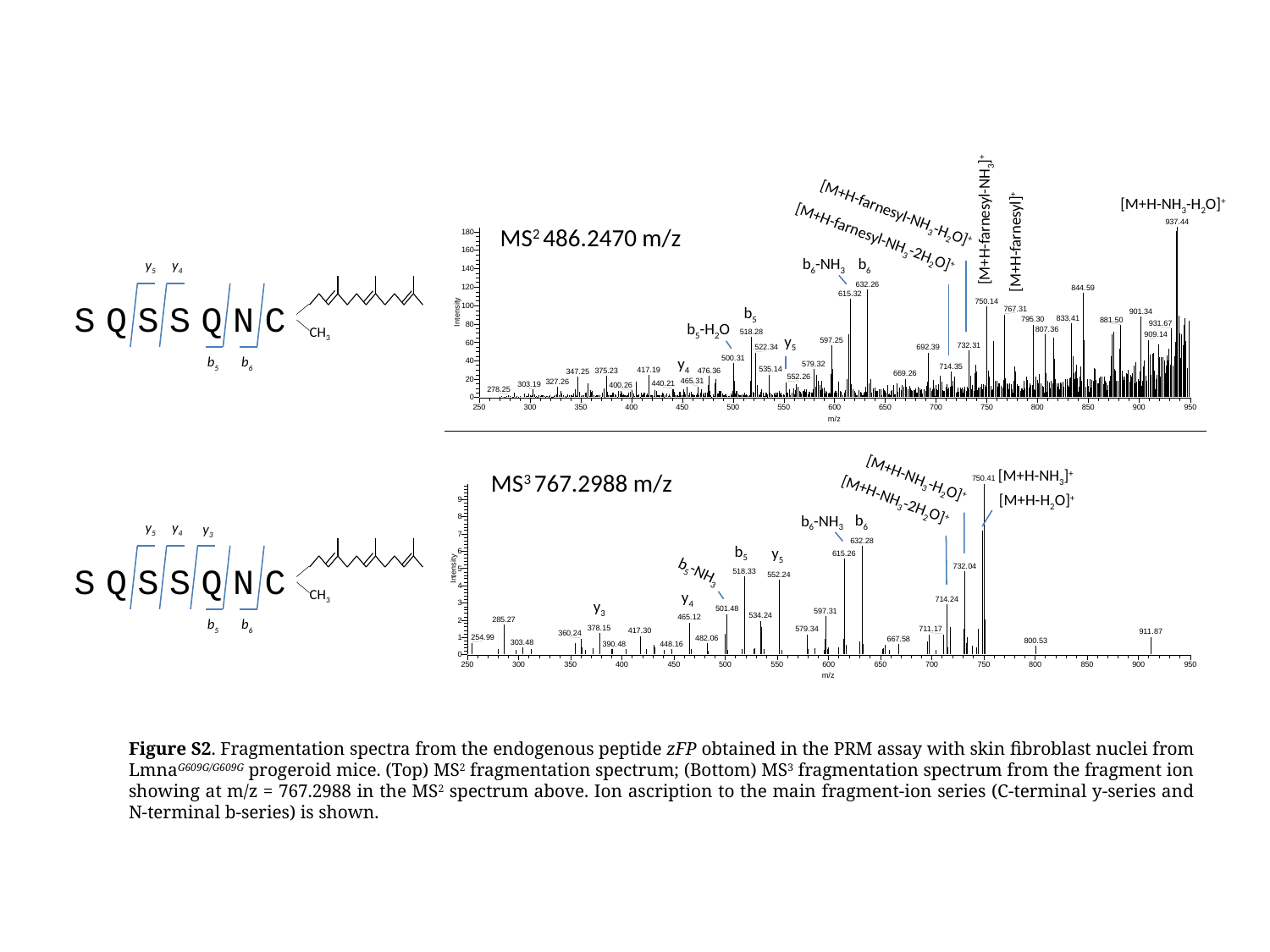

[M+H-NH3-H2O]+
[M+H-farnesyl-NH3]+
[M+H-farnesyl-NH3-H2O]+
[M+H-farnesyl]+
MS2 486.2470 m/z
[M+H-farnesyl-NH3-2H2O]+
b6
b6-NH3
y5
y4
S Q S S Q N C
b5
b5-H2O
CH3
y5
b5
b6
y4
[M+H-NH3]+
MS3 767.2988 m/z
[M+H-NH3-H2O]+
[M+H-H2O]+
[M+H-NH3-2H2O]+
b6
b6-NH3
y5
y4
S Q S S Q N C
CH3
b5
b6
y3
b5
y5
b5-NH3
y4
y3
Figure S2. Fragmentation spectra from the endogenous peptide zFP obtained in the PRM assay with skin fibroblast nuclei from LmnaG609G/G609G progeroid mice. (Top) MS2 fragmentation spectrum; (Bottom) MS3 fragmentation spectrum from the fragment ion showing at m/z = 767.2988 in the MS2 spectrum above. Ion ascription to the main fragment-ion series (C-terminal y-series and N-terminal b-series) is shown.
