# Supplementary material for: Quantification of Farnesylated Progerin in Hutchinson-Gilford Progeria Patient Cells by Mass Spectrometry
Source: Int J Mol Sci. 2022 Oct 3;23(19):11733. doi: 10.3390/ijms231911733 (PMC9569443; doi:10.3390/ijms231911733)
Supplement: Supplementary file 1 [file ijms-23-11733-s001.zip › ijms-1916264-supplementary/Supplementary Methods.pdf]

SUPPLEMENTARY METHODS

1. ETHICS STATEMENT

All animal works have been conducted according to Directive 2010/63EU and Recommendation 2007/526/EC regarding the protection of animals used for experimental and other scientific purposes, enforced in Spanish law under Real Decreto 1201/2005.

2. MATERIALS

2.1. Mouse cells and tissues

2.1.1. Mouse skin fibroblasts. Mouse adult fibroblasts were isolated from the ears of 15-week-old wild-type, and from progeroid homozygous *Zmpste24*$^{-/-}$ and *Lmna*$^{G609G/G609G}$ mice as described previously [27]. Clumped cells were disrupted by pipetting and further filtered through a 45 µm cell strainer to obtain single cell suspensions. After 4-5 cell passages, the primary fibroblasts were immortalized by retroviral transduction of SV40 large T antigen as described [28] and further maintained in high-glucose DMEM broth (Gibco) supplemented with 10% FBS (Gibco).

2.1.2. Mouse liver and heart tissue. Liver and heart tissues from 15-week-old wild-type mice were dissected, washed with PBS and immediately snap-frozen in liquid nitrogen.

2.2. Human cells

2.2.1. Human skin fibroblasts. Primary skin fibroblasts from four healthy individuals and four clinically affected HGPS patients carrying the typical c.1824C>T (p. Gly608Gly) mutation were obtained from the Progeria Research Foundation Cell and Tissue Bank. These fibroblasts were grown asynchronously in high glucose DMEM broth (Gibco) supplemented with 15% FBS

(Gibco), 2 mM L-glutamine and 100 units/mL of penicillin/streptomycin until enough cell material was attained.

2.2.2. U-2 OS cell line. Human osteosarcoma U-2 OS cells (Sigma-Aldrich) were grown in DMEM broth (Gibco) supplemented with 10% FBS (Gibco), 2 mM L-glutamine and 100 units/mL of penicillin/streptomycin.

2.2.3. Human lymphocytes. Human Jurkat-derived J77 (TCR Vαl.2 Vβ8) T-cells were cultured in RPMI 1640 broth (Gibco) supplemented with GlutaMAX–I, 25 mM HEPES and 10% FBS (Gibco).

2.2.4. Human white blood cells. White blood cells isolated from 4 mL of blood from three healthy individuals and three clinically affected HGPS patients carrying the typical c.1824C>T (p. Gly608Gly) mutation were provided by the Progeria Research Foundation Cell and Tissue Bank.

2.2.5. Synthetic peptides. The following synthetic peptides (JPT Peptide Technologies, Berlin, Germany) were used in the targeted precursor-reaction monitoring (PRM) LC-MS/MS assay (Fig. 6 of the manuscript) described in Subsection 5.2.3 below:

i. Synthetic, isotopically labelled internal control peptide, *IC** (TVLCGTCGQPADK, with $^{13}C_6$,$^{15}N_2$-Lys and two carbamidomethylated Cys);
ii. Synthetic, isotopically labelled lamin A peptide, *LA** (SVGGSGGGSFGDNLVTR, with $^{13}C_6$,$^{15}N_4$-Arg);
iii. Synthetic, isotopically labelled human progerin farnesylated peptide *hFP** (ASASGSGAQSPQNC, with $^{13}C_3$,^{15}N N-terminal Ala and O-methylated, farnesylated Cys).

3. PREPARATION OF PROTEIN EXTRACTS

3.1. Protein extracts for LC-MS/MS analyses. Nuclear fractions were prepared from whole cell pellets based on a non-ionic detergent-based procedure as described [29]. Frozen mouse tissues were ground with a mortar and further homogenized with a Polytron tissue grinder. Tissue, whole cell and nuclei extracts were prepared by boiling for 5 min in high-SDS lysis buffer (50 mM Tris-HCl pH 6.8, 4% SDS, 10 mM DTT). Then the lysates were centrifuged at 12,000 rpm at 4°C for 15 s and the protein concentration in the supernatant fraction was measured with the RC/DC protein assay kit (BioRad) according to the manufacturer's instructions.

3.2. Protein extracts for Western blot analysis. Cell extracts were prepared in low SDS lysis buffer (150 mM NaCl, 10 mM Na_2HPO_4, 2 mM KH_2PO_4, 2.7 mM KCl, 50 mM EDTA, 2.5 mM EGTA, 0.1% SDS, 1% NP-40, 0.5% sodium deoxycholate supplemented with protease inhibitors (Roche)) and protein concentration was assessed by the Bradford assay using BSA as a standard (Protein Assay Kit, Bio-Rad).

4. PROTEIN DIGESTION

The protein extracts obtained in Subsection 3.1 were trypsin-digested separately using the one-step in-gel digestion protocol we described previously [30]. Briefly, the protein extracts (200 µg), were loaded onto 1-cm wide wells of a conventional SDS-PAGE gel (0.5 mm-thick, 4% stacking, and 10% resolving). The run was stopped as soon as the front entered 2 mm into the resolving gel, so that the whole proteome became concentrated in the stacking/resolving gel interface. The unseparated protein bands were visualized by Coomassie staining, excised, cut into pieces, treated with DTT (Sigma-Aldrich) and iodoacetamide (Sigma-Aldrich) and digested overnight at 37 °C with trypsin (Promega, Madison, WI, USA) at 10:1 protein:trypsin

(w/w) ratio. The resulting tryptic peptide solutions were acidified with TFA (Sigma-Aldrich) and spiked with the isotopically labelled synthetic peptides *IC**, *LA** and *hFP** (Subsection 2.2.5 above) when necessary, after which the peptide mixtures were desalted in RP C-18 extraction cartridges (Oasis, Waters, Milford, MA, USA) and dried-down.

5. LC-MS/MS ANALYSES

5.1. Shotgun LC-MS/MS analysis. High-resolution shotgun analysis of peptides was carried out on a Ultimate 3000 nano-HPLC apparatus (Dionex, Sunnyvale, CA, USA) coupled to an orbital ion trap mass spectrometer (LTQ-Orbitrap XL, Thermo Scientific, San Jose, CA, USA). Peptides (30 theoretical µg) were suspended in 0.1% formic acid and then separated in a home-made C-18 RP nano-column (100 µm I.D., 45 cm) using a continuous gradient consisting of 8-31% B for 180 min and 31-90% B for 2 min (B = 90% acetonitrile, 0.1% formic acid) at 300 nL/min. Peptides were ionized using a Picotip emitter nanospray needle (New Objective, Woburn, MA, USA). Each MS run consisted of enhanced FT-resolution spectra (60,000 resolution) in the 390–1,200 m/z range followed by data-dependent MS/MS spectra of the five most intense parent ions acquired along the chromatographic run. The AGC target value in the Orbitrap for the survey scan was set to 1,000,000. Fragmentation in the linear ion trap was performed by CID at 35% normalized collision energy with a target value of 10,000 ions. The full target was set to 30,000, with 1 microscan and a 150 ms injection time, and the dynamic exclusion was set to 1 min.

5.2. Targeted PRM LC-MS/MS assays

5.2.1. PRM analysis for the relative quantitation of lamin A and progerin in mouse samples. Targeted LC-MS/MS analyses of mouse samples were carried out by high-resolution PRM on a Ultimate 3000 nano-HPLC apparatus (Dionex) coupled to an orbital ion trap mass

spectrometer (LTQ-Orbitrap XL, Thermo Scientific). The peptides were separated in a home-made C-18 RP nano-column (100 µm I.D., 45 cm) using a 300 nL/min flow and a continuous gradient consisting of 5–45% B for 120 min and 45–90% B for 2 min (B = 90% acetonitrile, 0.1% formic acid). Peptides were ionized using a Picotip emitter nanospray needle (New Objective). Each MS run consisted of enhanced FT-resolution spectra (15,000 resolution) in the 150–1,600 m/z range followed by data-independent acquisition of MS^2 or MS^3 spectra from the precursor ions displayed in Table 1. This cycle of data-independent full MS precursor and fragment scans was repeated along the chromatographic run.

5.2.2. PRM analysis for the relative quantitation of lamin A and progerin in human samples. For the relative quantitation of human lamin A and progerin, samples were analyzed by high-resolution PRM on an Easy nLC 1000 nano-HPLC apparatus (Thermo Scientific) coupled to a hybrid ion trap-orbitrap mass spectrometer (Orbitrap Elite, Thermo Scientific). The peptides were separated in a C-18 RP nano-column (75 µm I.D., 50 cm, Acclaim PepMap100, Thermo Scientific) using a 200 nL/min flow and a continuous gradient consisting of 5–45% B for 120 min and 45–90% B for 2 min (B = 90% acetonitrile, 0.1% formic acid). Peptides were ionized using a Picotip emitter nanospray needle (New Objective). Each MS run consisted of enhanced FT-resolution spectra (15,000 resolution) in the 150–1,600 m/z range followed by time-scheduled data-independent acquisition of MS^2 or MS^3 spectra from the precursor ions displayed in Table 2. Every cycle of data-independent full MS precursor and fragment scans was repeated along the corresponding time segment.

5.2.3. PRM analysis for the absolute quantitation of lamin A and progerin in human samples. For the absolute quantitation of human lamin A and progerin, samples were analyzed by high-resolution PRM on an Easy nLC 1000 nano-HPLC apparatus (Thermo

Scientific) coupled to a hybrid ion trap-orbitrap mass spectrometer (Orbitrap Elite, Thermo Scientific). The peptides were separated in a C-18 RP nano-column (75 µm I.D., 50 cm, Easy-spray C18, Thermo Scientific) using a 200 nL/min flow and a continuous gradient consisting of 5–45% B for 120 min and 45–90% B for 2 min (B = 90% acetonitrile, 0.1% formic acid). Each MS run consisted of enhanced FT-resolution spectra (15,000 resolution) in the 150–1,600 m/z range followed by time-scheduled data-independent acquisition of MS^2 spectra from the precursor ions displayed in Table 3. To optimize sensitivity, given that the behavior of the surrogate peptides was already known from the above-described MS^3-based PRM assays (Subsections 5.2.1 and 5.2.2), this PRM assay, which makes use of internal peptide standards, relied on MS^2 quantification, which resulted in an increased number of MS^2 fragmentations per unit time. Every cycle of data-independent full MS precursor and fragment scans was repeated along the corresponding time segment. The linear range, limit of detection, and limit of quantification of the PRM assay, which were assessed using tryptic digests of human skin fibroblasts from healthy donors spiked with varying amounts of the isotopically labelled synthetic peptides *IC**, *LA** and *hFP** (Subsection 2.2.5 above), were as follows:

	*IC**	*LA**	*hFP**
Linear range / fmol	2 - 250	1 - 125	8 - 500
Lower limit of quantification / fmol	2	1	8
Lower limit of detection / amol	500	250	2000

6. PROTEIN IDENTIFICATION AND LC-MS/MS DATA ANALYSIS

Protein identification from shotgun LC-MS/MS scans was performed using the SEQUEST HT algorithm integrated in Proteome Discoverer 1.4 (Thermo Scientific). The spectra were searched against either a mouse or a human database (UniProtKB/Swiss-Prot 2013_02 Release plus mouse or human progerin, respectively). For peptide identification the probability ratio method was used [31], and the false discovery rate (FDR) of peptide

identifications was calculated by the refined method [32]. Xcalibur 2.2 (Thermo Fisher Scientific) was used to obtain the extracted ion chromatograms (XICs) of the fragment ions indicated on Tables 1, 2, and 3 using 5 ppm tolerance.

7. WESTERN BLOT ANALYSIS

The protein extracts (50 µg) obtained in Subsection 3.2 were mixed with Laemmli sample buffer (62.5 mM Tris–HCl, pH 6.8, 2.3% SDS, 10% glycerol, 5% β-mercaptoethanol, 0.005% bromophenol blue), boiled for 10 min and resolved on 10% SDS–polyacrylamide gels. Proteins were then transferred to PVDF membranes using the iBlot® Dry Blotting system (Invitrogen), after which the membranes were incubated for 1 h at room temperature in blocking buffer (5% (w/v) non-fat dried milk in TBS–0.2% (v/v) Tween-20) followed by overnight incubation at 4 °C with primary antibodies diluted in blocking buffer. Primary antibodies were purchased from Sigma (anti-actin, ref. A2066) and Santa Cruz Biotech. (anti-lamin A/C, ref. sc-6214). After washing with TBS–0.2% Tween-20, the membranes were incubated with HRP-conjugated secondary antibodies (Santa Cruz Biotech.) for 1 h at room temperature. The specific proteins were then visualized by enhanced chemiluminescence (GE Healthcare).

Supplementary References

27. Varela, I.; Cadinanos, J.; Pendas, A.M.; Gutierrez-Fernandez, A.; Folgueras, A.R.; Sanchez, L.M.; Zhou, Z.; Rodriguez, F.J.; Stewart, C.L.; Vega, J.A.; et al. Accelerated ageing in mice deficient in Zmpste24 protease is linked to p53 signalling activation. *Nature* **2005**, *437*, 564-568, doi:10.1038/nature04019.
28. Tevethia, M.J.; Ozer, H.L. SV40-mediated immortalization. *Methods Mol Biol* **2001**, *165*, 185-199.
29. Dignam, J.D.; Lebovitz, R.M.; Roeder, R.G. Accurate transcription initiation by RNA polymerase II in a soluble extract from isolated mammalian nuclei. *Nucleic Acids Res* **1983**, *11*, 1475-1489, doi:10.1093/nar/11.5.1475.
30. Bonzon-Kulichenko, E.; Perez-Hernandez, D.; Nunez, E.; Martinez-Acedo, P.; Navarro, P.; Trevisan-Herraz, M.; Ramos Mdel, C.; Sierra, S.; Martinez-Martinez, S.; Ruiz-Meana, M.; et al. A robust method for quantitative high-throughput analysis of proteomes by 18O labeling. *Mol Cell Proteomics* **2011**, *10*, M110 003335, doi:10.1074/mcp.M110.003335.
31. Martinez-Bartolome, S.; Navarro, P.; Martin-Maroto, F.; Lopez-Ferrer, D.; Ramos-Fernandez, A.; Villar, M.; Garcia-Ruiz, J.P.; Vazquez, J. Properties of average score distributions of SEQUEST: the probability ratio method. *Mol Cell Proteomics* **2008**, *7*, 1135-1145, doi:10.1074/mcp.M700239-MCP200.
32. Navarro, P.; Vazquez, J. A refined method to calculate false discovery rates for peptide identification using decoy databases. *J Proteome Res* **2009**, *8*, 1792-1796, doi:10.1021/pr800362h.
